# Sulforaphane from Brassica Oleracea Induces Apoptosis in Oral Squamous Carcinoma Cells via p53 Activation and Mitochondrial Membrane Potential Dysfunction

**DOI:** 10.3390/ph18030393

**Published:** 2025-03-11

**Authors:** Pooja Narain Adtani, Sura Ali Ahmed Fuoad Al-Bayati, Walid Shaaban Elsayed

**Affiliations:** 1 Department of Basic Medical and Dental Sciences, College of Dentistry, Gulf Medical University, Ajman 4184, United Arab Emirates; 2 Department of Diagnostic and Surgical Dental Sciences, College of Dentistry, Gulf Medical University, Ajman 4184, United Arab Emirates; dr.sura@gmu.ac.ae; 3 Department of Oral Biology, College of Dentistry, Suez Canal University, Ismailia 41522, Egypt

**Keywords:** apoptosis, oral squamous cell carcinoma, sulforaphane

## Abstract

**Background/Objectives:** Oral squamous cell carcinoma (OSCC) is a significant global health concern, necessitating the development of novel treatment strategies. The present study investigated the in vitro anticancer activity of sulforaphane (SFN), an isothiocyanate derived from Brassica oleracea, on the OECM-1 human oral squamous carcinoma cell line. **Methods:** OECM-1 cells were cultured and exposed to a range of SFN concentrations. To assess the cell viability and determine the half maximal inhibitory concentration (IC50) of SFN following 24 h of treatment, an MTT assay was performed. Apoptosis was evaluated using AO/PI staining, a TUNEL assay, Annexin V-FITC analysis, and a DNA fragmentation assay. Changes in the mitochondrial membrane potential were analyzed using a JC-1 staining assay. A Western blot assay was performed to assess the expression levels of apoptosis-associated proteins (Bax, Bcl2, caspase-3, caspase-9, PARP, Smad-4, p53, cytochrome c, and GAPDH). Cell cycle analysis was performed to validate the apoptotic findings. **Results:** The IC50 concentration of SFN was 5.7 µM. The apoptotic assays demonstrated an effective induction of apoptosis in the OECM-1 cells. Western blot analysis demonstrated the dose-dependent upregulation of p53, caspase-3, caspase-9, PARP, cytochrome c, and Bax and the downregulation of the anti-apoptotic proteins Bcl-2 and Smad-4 after SFN treatment. **Conclusions:** The data obtained indicate that SFN has significant potential to induce apoptosis in OECM-1 cells by disrupting mitochondrial function and modulating apoptotic pathways. The outcomes of our research indicate SFN’s potential as a viable treatment drug for OSCC.

## 1. Introduction

Head and neck squamous cell carcinoma (HNSCC) ranks as the sixth most common cancer globally, with oral squamous cell carcinoma (OSCC) accounting for approximately 60% of these tumors. OSCC is a malignancy that develops from different areas of the oral cavity namely tongue, hard palate, alveolar mucosa, buccal mucosa, and lip [1,2,3]. It most commonly affects middle-aged and elderly individuals, although cases have also been reported in young adults [4]. Various factors, such as genetic susceptibility, mutations, tobacco use, areca nut chewing, excessive alcohol intake, extended sun exposure, and poor dental hygiene, have been implicated as causes of OSCC [3,5,6]. A subgroup of OSCC is associated with human papillomavirus (HPV), namely HPV-16 and HPV-18. Viral oncoproteins that interfere with regular cell cycle control are expressed due to the integration of the virus into the host genome [7]. Additionally, a diet deficient in antioxidants and vitamins such as A, C, E, and folate might impair cellular repair and immune system performance, leading to the development of OSCC [8,9]. Timely identification and treatment can help increase survival rates. Preventive measures and early diagnosis can be supported by routine oral examinations and avoiding recognized risk factors [3].

The standard treatment for OSCC involves surgery, radiation therapy, and chemotherapy [3]. Depending on the characteristics of the malignancy, these treatments may be performed alone or in combination. A multidisciplinary approach is usually necessary for the effective management of OSCC, involving not only oncologists but also nutritionists to address dietary needs, speech therapists to aid in functional recovery, physiotherapists, and immune therapists to support immune function and general health. This multidisciplinary approach aids in addressing several facets of patient care, including quality of life, rehabilitation, and cancer therapy [3,10,11,12]. Despite the availability of various treatment modalities, and due to the accompanying complications, the 5-year survival rate has remained at approximately 50% [3].

Sulforaphane (SFN) is a phytochemical classified as a sulfur-containing isothiocyanate. It was first discovered and isolated in 1959 and is primarily present in cruciferous vegetables including broccoli, cabbage, and cauliflower. In plants, it is found as glucosinolates, which are further converted to SFN via the myrosinase-catalyzed process during extraction. Upon ingestion, SFN is primarily absorbed in the jejunum, and its bioavailability widely ranges from 1% to 40%, with maximum absorption occurring within the first 8 h post-dose. The conversion of glucoraphanin to SFN largely depends on the patient’s gut microbiome, along with factors such as diet, genetics, and metabolic differences. Although SFN, being a naturally available compound, is ‘generally regarded as safe’, studies have reported a few side effects, such as abdominal pain, gastritis, and diarrhea, upon excessive consumption of broccoli due to its high fiber content [13,14,15]. 

SFN has only recently gained attention because of its diverse biological properties including its antimicrobial, antioxidant, antitumorigenic, antiangiogenic, and anti-inflammatory effects [6,16]. SFN has been widely explored for its anticancer properties in prostate, breast, liver, gastric, and lung cancers [14]. In a study conducted by Yao et al., it was observed that treatment with SFN in the Tca8113 human tongue squamous carcinoma cell line resulted in the downregulation of hypoxia-inducible factor 1 alpha (HIF-1α) expression levels. According to this study, SFN may have an impact on how cancer cells respond to low oxygen levels. Furthermore, Chao et al. discovered that SFN had an inhibitory effect on the expression of cyclo-oxygenase-2 (COX-2) in both mouse xenograft models and OSCC cell lines. By inhibiting COX-2, SFN demonstrates its potential to manipulate important signaling pathways and trigger apoptosis, effectively causing the death of cancer cells [17,18].

Additionally, in comparison to cancer therapies, such as chemotherapy and radiation, studies have reported SFN to be non-oxidizable, less toxic, and well tolerated upon administration, making it suitable for clinical trials [15]. Sharma et al. reported that SFN, when administered synergistically with gemcitabine in HeLa cells, exhibited concentration-dependent anticancer activity compared to gemcitabine alone, thereby improving the therapeutic index for treating cervical cancer [19].

However, further studies are necessary to investigate and validate the potential therapeutic applications of SFN in the treatment of OSCC. The purpose of our study was to assess the potential anticancer effects of SFN and understand the apoptotic pathways in the human squamous cell carcinoma cell line (OECM-1) via the caspase-dependent pathway.

## 2. Results

### 2.1. Effects of SFN on OECM-1 Cells and Its Anticancer Activity

**Figure 1:**
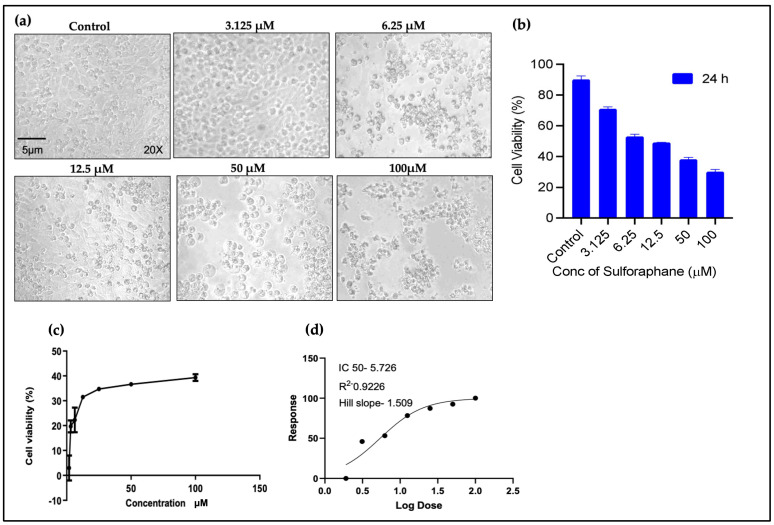
Cell viability assay results of OECM-1 cells with SFN treatment for 24 h. (**a**) Cells were visualized using phase-contrast microscope magnification at 20× at different SFN concentrations (3.125 μM, 6.25 μM, 12.5 μM, 50 μM, and 100 μM). (**b**) Graphical representation of cell viability after SFN induction. (**c**,**d**) Log dose calculation shows an IC50 value of 5.7 μM.

The MTT assay results demonstrate a distinct correlation between cell viability and the administered dosage (Figure 1a). Following a 24 h incubation period with SFN, almost 50% of the cells remained viable at 6.25 µM (SFN), as depicted in Figure 1b. Furthermore, the non-linear regression curve fit indicates the quantity of viable and non-viable cells. According to the log dose calculation, an IC50 of 5.7 µM SFN is shown in Figure 1c,d.

**Figure 2:**
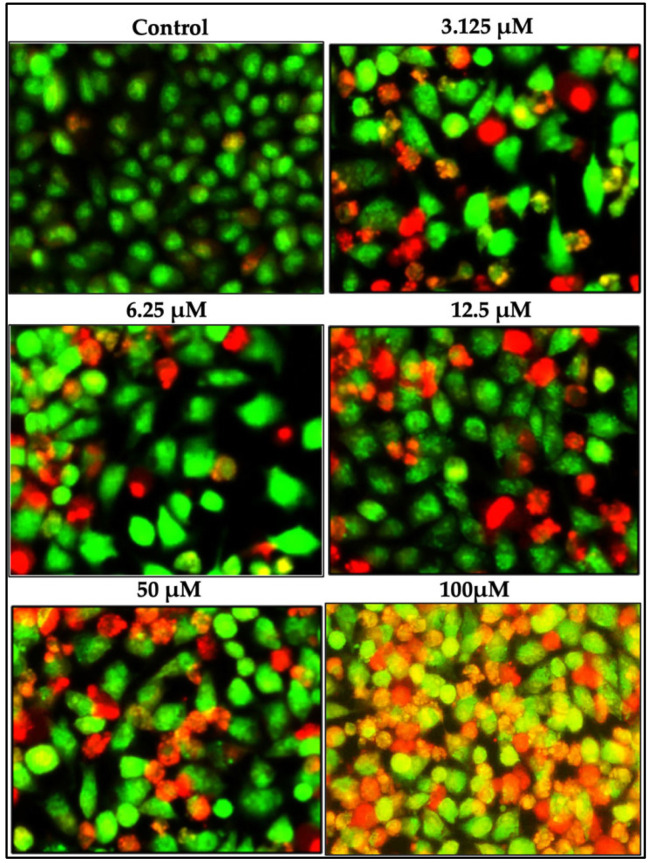
Acridine orange/propidium iodide (AO/PI) staining to evaluate morphological changes during apoptosis induced by SFN at various concentrations; the control group received 0.1% dimethyl sulfoxide (DMSO). Scale bar = 5 μM.

Following the MTT assay, the impact of the SFN treatment on cellular apoptosis was evaluated by treating the OECM-1 cells with different concentrations of SFN for 24 h. AO is capable of staining both live and dead cells, giving them a green color. On the other hand, PI is used to stain cells that have lost their membrane integrity, resulting in a red color. Green staining indicates the presence of viable cells, while reddish or orange staining indicates cells in the early or late stages of apoptosis. Uniform green live cells with normal nuclei were observed in the control group, while the SFN-treated group showed cells stained with orange and red. Figure 2 demonstrates the effective apoptotic activity of SFN at various concentrations (3.125 μM, 6.25 μM, and 12.5 μM). The above results demonstrate that SFN effectively induced apoptosis in the oral cancer cells.

### 2.2. Evaluation of Apoptosis in SFN-Treated OECM-1 Cells Using TUNEL Assay

**Figure 3:**
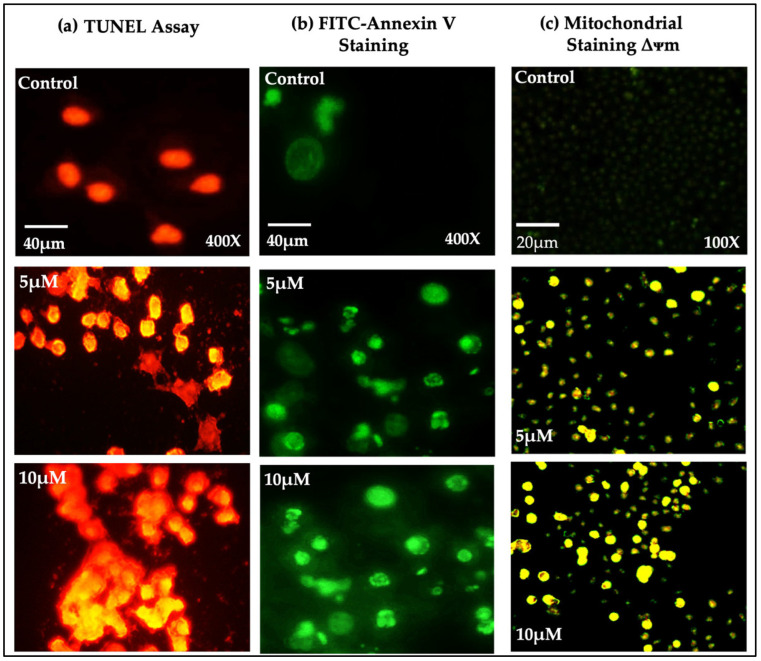
(**a**,**b**) Evaluation of apoptosis in 24 h SFN-treated (5 μM and 10 μM) OECM-1 cells using TUNEL and annexin V–FITC staining assay. Scale bar = 40 μM; magnification of 400×. (**c**) Effects of SFN (5 μM and 10 μM) on mitochondrial membrane potential of OECM-1 cells after 24 h treatment. Scale bar = 20 μM; magnification of 100×.

The assay results demonstrate the effects of treating OECM-1 cells with SFN (5 μM and 10 μM) for 24 h. The experimental results suggest a significant increase in the number of TUNEL-positive (apoptotic) cells, accompanied by nuclear disintegration and chromatin condensation. The confocal image (Figure 3a) confirms that the TUNEL-positive cells increased after treatment with SFN.

### 2.3. Evaluation of Apoptosis in SFN-Treated OECM-1 Cells Using FITC-Annexin Assay

FITC-annexin V/7-AAD (7-aminoactinomycin D) analysis was performed by treating the OECM-1 cells with 5 and 10 µM doses of SFN for 24 h. Notably, the rate of cellular apoptosis (early apoptosis) significantly increased in a dose-dependent manner in the cells treated with SFN compared to the control group, as shown by the percentage of cell death (Figure 3b). Moreover, according to the obtained results, it was found that SFN effectively triggered substantial cellular apoptosis in cancer cells.

### 2.4. Effects of SFN on Mitochondrial Transmembrane Potential in OECM-1 Cell Line Using JC-1 Dye

OECM-1 cells subjected to SFN at different concentrations (5 μM and 10 μM) exhibited distinct indications of apoptosis, as demonstrated by the penetration of JC-1 dye. However, the impairment in the formation of J-aggregates can be attributed to the increased permeability of the mitochondrial membrane and a decrease in the negative charge within the mitochondria. Under normal conditions, healthy mitochondria produce J-aggregates, resulting in red fluorescence; however, in these cells, mitochondrial dysfunction obstructed this process. The JC-1 dye preserved its original monomeric configuration, resulting in the emission of green fluorescence. This suggests a decrease in the mitochondrial membrane potential, a defining characteristic of early apoptosis. The observation of green fluorescence indicates a significant disruption in mitochondrial function following SFN treatment, thus reinforcing the involvement of mitochondria in the apoptotic mechanism (Figure 3c).

### 2.5. DNA Fragmentation Analysis

**Figure 4:**
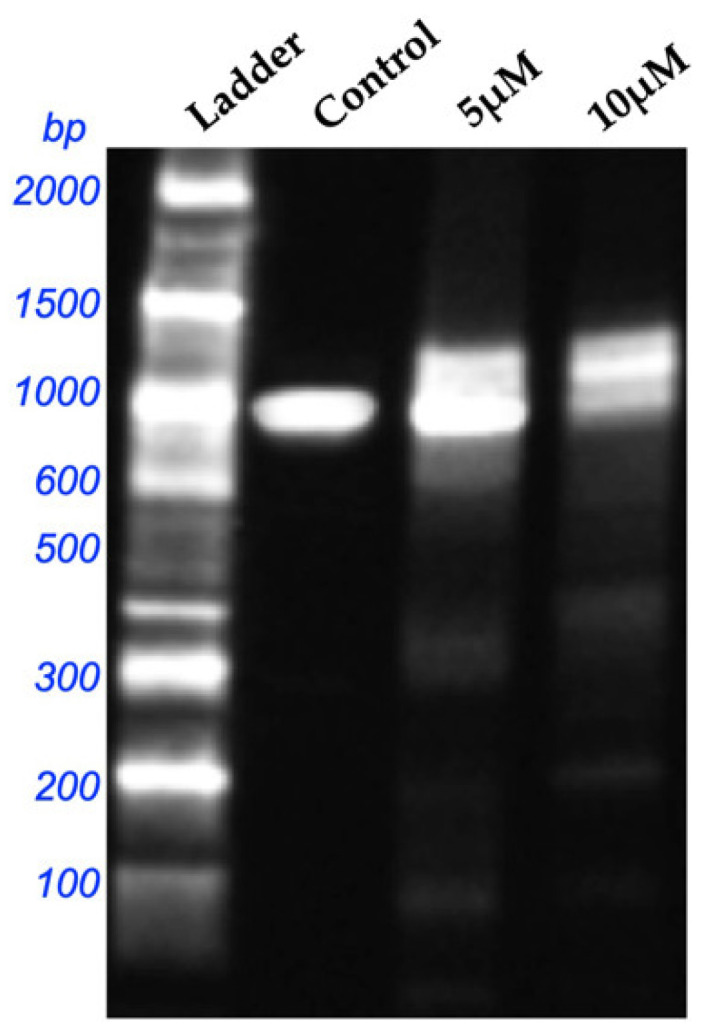
DNA fragmentation analysis of OECM-1 cells treated with 5 μM and 10 μM of SFN compared to control.

The ladder band comprises DNA fragments of a known size, serving as a reference to estimate the sizes of the bands in the other lanes. The control group should have minimal DNA fragmentation. The 5 μM and 10 μM bands represent the concentrations of SFN investigated. The ladder shows distinct patterns of bands of many sizes. The control band had a comparatively light band at around 1000 bp, indicating negligible fragmentation in the control group. Both the 5 μM and 10 μM bands exhibited a significant increase in the intensity of bands at 1000 bp and lower, signifying augmented DNA breakage with elevated SFN doses. These results indicate that SFN induced DNA fragmentation in a concentration-dependent way. The heightened intensity of the lower-molecular-weight bands in the 5 μM and 10 μM lanes indicates DNA degradation (Figure 4).

### 2.6. Flow Cytometry Analysis

**Figure 5:**
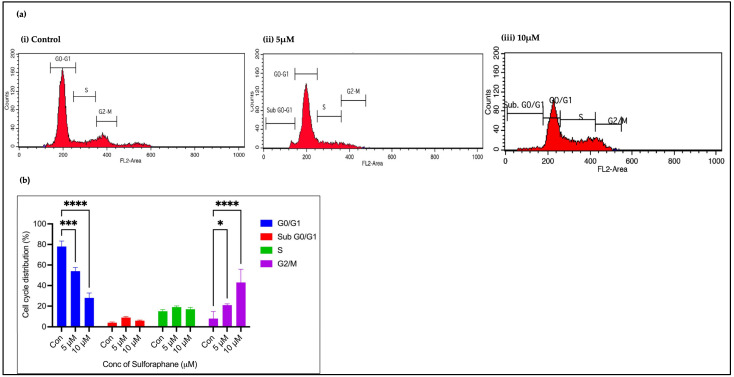
Growth-inhibitory effects of SFN on OECM-1 cells. (**a**) The cell cycle phase distribution of OECM-1 cells after treatment with 5 μM and 10 μM of SFN compared to the control. (**b**) Graph illustrating the percentage of OECM-1 cells in various phases of the cell cycle after 5 μM and 10 μM of SFN treatment compared to the control (* *p* ≤ 0.05, *** *p* ≤ 0.001, **** *p* ≤ 0.0001).

The histogram displays the standard distribution of the cell cycle in the untreated (control) cells. The majority of the cells were in the G0-G1 phase, compared to the S and G2-M phases, implying that most of them were in a state of rest or early development. The second and third groups of cells were treated with 5 μM and 10 µM concentrations of SFN, respectively. The control graph shows that the sub-G0-G1 peak represents cells with sub-G1 DNA that were undergoing apoptosis. The G0-G1 peak indicates the presence of cells with 2N DNA, whereas the S phase peak, situated between G0-G1 and G2-M, represents the process of DNA replication. The G2-M peak indicates the presence of cells containing 4N DNA, which were in the process of preparing for division. The sub-G0-G1 peak in the 5 µM SFN-treated group suggests cells with decreased DNA, signifying apoptosis. The S phase peak signifies that the cells engaged in DNA replication, while the G2-M peak indicates that the cells with 4N DNA prepared for division, which was similar to the control group. In the 10 µM SFN-treated group, the sub-G0-G1 peak indicates apoptotic cells. The increased G0-G1 peak indicates that the cells are in a quiescent or early developmental state, while the enlarged S phase peak signifies active DNA replication. The G2-M peak signifies cells with duplicated DNA (4N) (Figure 5a) primed for division. The graph explains the percentages of cells in the different phases of the cell cycle with and without SFN treatment (Figure 5b). The graph illustrates that SFN treatment at both the 5 µM and 10 µM concentrations elevated cell mortality, as evidenced by the increase in the sub-G0-G1 peak and the reduction in G0-G1. In comparison to the 5 µM treatment, the 10 µM dose induced a more significant shift toward apoptosis characteristics of DNA fragmentation.

### 2.7. Western Blot Analysis

**Figure 6:**
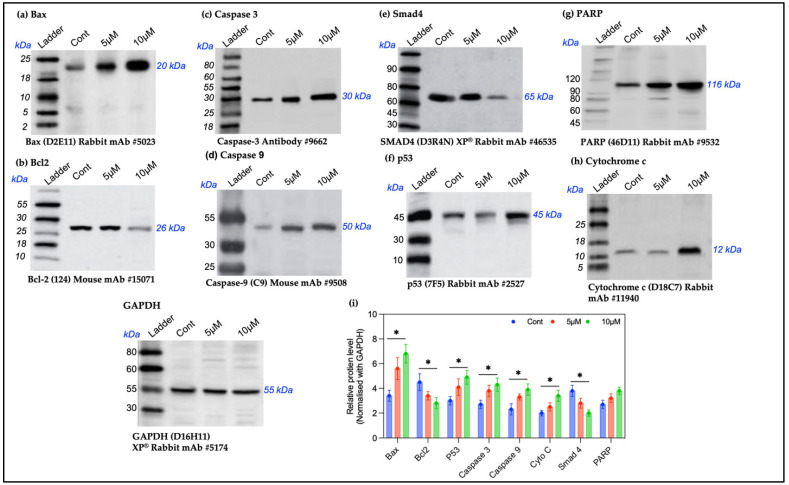
(**a**–**h**) Expression levels of pro-apoptotic markers (Bax, caspase-3, and caspase-9), an anti-apoptotic marker (Bcl2), tumor suppressor genes (p53 and Smad4), PARP, and cytochrome c cells after SFN induction at the designated concentrations (5 μM and 10 μM) against GAPDH as a control using Western blotting. (**i**) Graphical representation of Bax, caspase-3, caspase-9, Bcl2, p53, Smad4, PARP, and cytochrome c in OECM-1 cells after SFN induction at the designated concentrations using Western blotting (* *p* < 0.05).

To evaluate the effect of SFN treatment on apoptosis-related protein expression in OECM-1 oral cancer cells, Western blot analysis was performed. The results indicate that the SFN treatment led to concentration-dependent effects on the Bcl-2 protein family, influencing the growth-inhibiting activity of SFN in OECM-1 cells. Specifically, the pro-apoptotic protein Bax showed significantly increased expression in OECM-1 cells treated with 5 μM and 10 μM of SFN compared to the untreated control group (Figure 6a). Meanwhile, the expression of the anti-apoptotic protein Bcl-2 was downregulated in the SFN-treated groups, particularly in the 10 μM SFN-treated cells (Figure 6b). Furthermore, this study observed increased expression levels of caspase-3, caspase-9, PARP, and cytochrome c in the SFN-treated groups compared to the control group (Figure 6c,d,g,h). This indicates an elevation in apoptosis. Additionally, the treatment with SFN upregulated the expression of the tumor suppressor gene p53, suggesting that SFN induced apoptosis by causing DNA damage (Figure 6f). Moreover, the expression of Smad4 was significantly downregulated in the SFN-treated groups compared to the untreated group (Figure 6e). Our study observed that SFN treatment affected the expressions of Bcl-2 family proteins, leading to cell death in OECM-1 oral cancer cells. The observed alterations in the protein and mRNA expression levels indicate the significant impact of Bcl-2 family proteins on the cell death process induced by SFN. The results indicate a dose-dependent effect on the protein expression, with increased levels of pro-apoptotic proteins and decreased levels of anti-apoptotic proteins in the SFN-treated groups. The relative protein levels normalized using GAPDH are depicted in Figure 6i.

## 3. Discussion

OSCC is the most common form of cancer that affects the head and neck region. Tobacco and alcohol are significant risk factors in the majority of OSCC cases, followed by viral infections, such as HPV, which contribute to the rising incidence of OSCC among young individuals, especially non-smokers [20,21,22]. Considering the constant demand for a novel therapeutic agent for blocking the growth of cancer cells, there is much interest in the antiproliferative abilities of natural compounds. Previous studies have shown that compounds found in Brassica vegetables, such as indoles and isothiocyanates, possess anticancer properties by affecting certain cellular pathways involved in apoptosis [23]. SFN, an isothiocyanate found naturally in broccoli, cauliflower, and cabbage, is known to inhibit the growth of cancer cells [24]. Despite extensive research on breast, pancreatic, gastric, lung, and prostate cancer, and very few studies on HNSCC, a comprehensive understanding of how SFN precisely exhibits anticancer effects in the OECM-1 oral cancer cell line will aid in targeting the signaling pathways involved in tumorigenesis [25,26,27].

Devi et al. (2012) examined the effect of SFN on cell growth using the human epithelial (Hep-2) cell line, reporting that apart from its cytotoxic effect, SFN also induced the apoptosis of Hep-2 cells [28]. Our results corroborate these findings, demonstrating that SFN effectively reduced the cellular viability of the OECM-1 cell line in a dose-dependent manner, with only 50% viability at a concentration of 6.25 μM, determining the IC50 as 5.7 μM via a log dose calculation.

Kntayya et al. (2018) reported the induction of apoptosis in hepatocellular carcinoma (HepG2) cells after treatment with SFN using TUNEL and AO/PI staining in a time-dependent manner [29]. In our study, AO/PI staining in OECM-1 cells exhibited characteristic apoptotic morphological changes induced by SFN at various concentrations (3.125 μM, 6.25 μM, and 12.5 μM), including the TUNEL assay, which demonstrated TUNEL-positive cells with nuclear disintegration and chromatin condensation, closely aligning with the observations of Kntayya et al. These results are further supported by the DNA fragmentation observed in the OECM-1 cells after treatment with 5 μM and 10 μM of SFN as compared to the control. Additionally, in our study, the annexin V assay demonstrated a decrease in the number of viable cells after treatment with SFN at concentrations of 5 μM and 10 μM, indicating that SFN effectively triggered substantial cellular apoptosis in the OECM-1 cells.

Our results align with a study by Yasuda et al. (2019), which demonstrated that the combination of SFN and co-culture with *Lactobacillus*-treated PBMCs significantly decreased the mitochondrial membrane potential in human colon cancer HCT116 and SW480 cells, indicating an increase in apoptosis [30]. As mentioned by Yasuda et al. (2019), we also performed a JC-1 staining assay to detect the effects of SFN on the mitochondrial transmembrane potential in the OECM-1 cell line. Our findings reveal that a lower penetration rate of the JC-1 dye occurred due to the increase in the mitochondrial membrane permeability, leading to a decrease in the mitochondrial membrane potential. As a result, the formation of J-aggregates was inhibited, and the cells retained the original green fluorescence stain, indicating their transition from a healthy state to apoptotic conditions.

Flow cytometry was used to analyze the effects of SFN on the cell cycle distribution of OECM-1. The majority of the untreated (control) cells were in the G0–G1 phase, indicating that they were either in a resting state or in the early stages of growth. It is normal to observe cell dispersion. The synthesis of DNA in the S phase and the preparation for mitosis were reduced in proportion to the number of cells. The baseline distribution matched the typical cell cycle patterns of both normal and malignant cells [31]. The use of SFN altered the cell cycle distribution, suggesting its potential as an anticancer agent. The sub-G0-G1 population significantly increased in the groups treated with 5 μM and 10 μM of SFN, indicating enhanced cell death or apoptosis. Increasing the concentration to 10 μM showed a dose-dependent impact of SFN on cell death. The sub-G0-G1 peak indicates the breakdown of the DNA in apoptotic cells. The phase of cell cycle arrest varies depending on the type of cancer cell line being treated with SFN. In a study on ovarian cancer cell lines (MDAH 2774), SFN at 10 μM induced G1 arrest in a time-dependent manner [32]. SFN induces oxidative stress and mitochondrial dysfunction, which can trigger both intrinsic and extrinsic apoptosis.

The overexpression of Bax and the downregulation of Bcl-2 reinforce the apoptotic effects of SFN, with the cleavage of PARP and the activation of caspase-3 and caspase-9 demonstrating the participation of the caspase-mediated pathway. Similar observations were reported in pancreatic and gastric cancer cells, in which 5–20 μM of SFN induced apoptosis with decreases in Bcl2 and increases in the Bax and caspase-3 levels [33,34]. Cells treated with SFN were arrested in the G2-M phase, indicating that SFN regulates the cell cycle via a p53-dependent mechanism. This suggests that when DNA is damaged, p53 induces apoptosis and cell cycle arrest to preserve genomic integrity. Additionally, the downregulation of the tumor suppressor Smad4 in the SFN-treated cells points to a complex signaling mechanism responsible for SFN’s anticancer effects [35,36]. The findings of our study partially align with those of Kim JH et al., who reported that treatment with 20 and 40 μM of SFN for 12 h resulted in G2/M phase cell cycle arrest in KB and YD-10B human oral squamous carcinoma cells and was linked to a significant increase in p21 protein levels [37].

Our findings highlight the role of SFN in triggering apoptosis in OECM-1 oral cancer cells by releasing cytochrome c from the mitochondria, activating caspase-9 and caspase-3, and increasing p53 expression. These effects, alongside the decrease in anti-apoptotic Bcl-2 levels and the increases in pro-apoptotic Bax, caspase-3, caspase-9, cytochrome c, and PARP, underscore SFN’s therapeutic potential for treating OSCC by inducing cell cycle arrest in the G2-M phase through DNA damage response and apoptosis initiation.

## 4. Materials and Methods

### 4.1. Cell Culture and Treatment

The OECM-1 cell line, sourced from Sigma Aldrich, St. Louis, MO, USA, was cultured in Dulbecco’s modified Eagle’s medium (DMEM)/F-12 enriched with 10% fetal bovine serum (FBS) and 1% antimycotic–antibiotic. The cells were maintained at 37 °C in an atmosphere of 5% CO_2_ [38]. All cell culture reagents used were purchased from Invitrogen (Carlsbad, CA, USA).

### 4.2. Cell Viability Assay of OECM-1 Cells Treated with SFN

To assess cell viability, a calorimetric MTT 3-(4,5-dimethylthiazol-2-yl)-2,5-diphenyltetrazolium bromide dye reduction assay was performed using reagents from Sigma, St. Louis, MO, USA. OECM-1 cells (20,000 cells/well) were seeded in a 24-well plate and allowed to grow for 24 h. Subsequently, the cells were exposed to SFN at concentrations of 100 µM, 50 µM, 12.5 µM, 6.25 µM, and 3.125 µM for 24 h. After 24 h of incubation, the cell medium was replaced with 400 μL of 0.5 mg/mL MTT reagent for 3 h [39]. Isopropanol was used to dissolve the formazan crystals formed in the cells. To achieve this, the plate was wrapped in foil and shaken in an orbital shaker for 15 min. Following this, a microplate reader (Model 680, Bio-Rad, Hercules, CA, USA) was utilized to measure the OD at 570 nm. Then, the 50% inhibitory concentration (IC50) and its linear regression curve fit were calculated for the SFN drug.

### 4.3. Acridine Orange/Propidium Iodide Staining Assay

Cultured OECM-1 cells were treated with varying concentrations of SFN (100 µM, 50 µM, 12.5 µM, 6.25 µM, and 3.125 µM) for 24 h. Following incubation, the cells were stained with acridine orange (AO) and propidium iodide (PI) solution (Nexcelom Bioscience, Lawrence, MA, USA) for 40–45 min [40] to assess cell viability. The procedure included mixing a 1:1 AO/PI solution with 20:20 μL of the SFN-treated cells. Lastly, the stained OECM-1 cells were observed, and images of both the control and the SFN treated with the desired concentration were captured using fluorescence microscopy. The dye (AO/PI) for both cytoplasmic and nuclei staining showed the excitation of live cells (green) at 488 nm and that of the dead cell nuclei (red) at a 570 nm wavelength [41].

### 4.4. TUNEL Assay

A terminal deoxynucleotidyl transferase dUTP nick end labeling (TUNEL) assay was performed following the instructions provided by the manufacturer (Abcam: ab252888). Cells were cultured in 6-well plates and incubated in a growth medium supplemented with 5 μM of SFN. Then, the TUNEL reaction mixture was added to all wells, except for those with the background control and unstained cells. The cells were incubated overnight at room temperature. After the overnight incubation, the click reaction mixture was added to the cells, followed by 30 min of incubation at room temperature. Finally, the samples were analyzed for red fluorescence generated by TUNEL-positive cells using an inverted confocal microscope [42].

### 4.5. Annexin V Staining

Cells treated with SFN in a 6-well plate were trypsinized, washed with phosphate-buffered saline (PBS), and resuspended in a 1× binding buffer using the annexin V-FITC apoptosis detection kit (annexin V–fluorescein isothiocyanate; BD Biosciences Pharmingen, San Diego, CA, USA). A 100 µL aliquot of the cell suspension was transferred into polystyrene tubes, followed by the addition of 5 µL annexin V-FITC for staining. The samples were wrapped in foil and incubated at room temperature for 15 min. Subsequently, 400 µL of 1× binding buffer was added, and the cells were analyzed on a FACScan flow cytometer (Becton Dickinson) [43].

### 4.6. Mitochondrial Membrane Potential

5,5,6,6′-tetrachloro-1,1′,3,3′ tetraethylbenzimi-dazoylcarbocyanine iodide (JC-1) dye procured from Thermo Fisher (Cat: #T3168) was dissolved to a final concentration of 2.5 μM using 15% dimethyl sulfoxide (DMSO). The cells were then exposed to JC-1 dye and incubated in the dark at 37 °C for 15 min. Subsequently, the supernatant was discarded, and the cells were analyzed using a Leica SPE confocal microscope. Further emission spectra of the samples at 590 nm were measured as per the manufacturer’s protocol. Then, they were determined using an FL 6500 fluorescence spectrometer [44].

### 4.7. DNA Fragmentation Assay

A DNA fragmentation procedure was conducted using agarose gel electrophoresis. The genomic DNA from OECM-1 cells, both with and without SFN treatment, was isolated using the phenol–chloroform–isoamyl alcohol technique [45]. Electrophoresis was carried out on 1.5% agarose gel to observe the formation of DNA fragmentation. The results were visualized using the Gel Doc system and thoroughly documented.

### 4.8. Cell Cycle Analysis

Cells in 6-well plates were treated with 5 μM and 10 μM of SFN for 24 h. Following treatment, cells were trypsinized, washed with PBS, and fixed at −20 °C in cold 70% ethanol. The fixed cells were subsequently collected and stained with a solution containing 0.5 mL of 4 μg/mL of propidium iodide (PI), 0.5 mg/mL of RNase, and 1% Triton X-100, and incubated for 30 min at 4 °C. Based on the DNA content, the cell cycle distribution was analyzed using a FACScan flow cytometer (Becton Dickinson, Franklin Lakes, NJ, USA [46].

### 4.9. Western Blot

OECM-1 cells were treated with SFN at concentrations of 5 μM and 10 μM for 24 h. Following treatment, proteins were extracted using a radioimmunoprecipitation assay (RIPA) cell lysis buffer (SKU: AR0105-100, Boster Bio’s, Pleasanton, CA, USA) and kept on ice for at least 30 min. The cell lysates were then centrifuged at 12,000× *g* at 4 °C for 10 min, and the supernatant was transferred to a clean tube. Protein concentrations were estimated using the bicinchoninic acid (BCA) method, and equal amounts of total protein per lane were loaded and separated using sodium dodecyl sulfate–polyacrylamide gel electrophoresis (SDS-PAGE). Proteins were then transferred to polyvinylidene fluoride (PVDF) membranes.

The membranes were blocked with a solution containing 10% bovine serum albumin (BSA) powder in 0.05% Tris-buffered saline and Tween 20 (TBST) for 1 h at room temperature. They were subsequently incubated overnight at 4 °C with antibodies against Bax, Bcl2, caspase-3, caspase-9, PARP, Smad-4, p53, cytochrome c, and GAPDH [47]. After overnight incubation, the membranes were washed thrice and incubated with peroxidase-conjugated secondary antibodies for 2 h at room temperature. The blots were developed with Western Lightning Plus using enhanced chemiluminescence (ECL) reagents (PerkinElmer, Waltham, MA, USA), and their intensities were quantified using I Quant TL software 8.1 (GE Healthcare, Chicago, IL, USA).

### 4.10. Statistics

The data are presented as the mean values with standard deviations from three independent experiments. Statistical analysis was performed using a two-way ANOVA and Bonferroni’s post hoc test using Graph Pad Prism 9.0.

## 5. Conclusions

In summary, OECM-1 cells were used to assess the effectiveness of SFN in inhibiting tumor growth in human oral squamous carcinoma cells. The results of the MTT assay show that it has great potential, as the cell viability decreased in a dose-dependent manner, indicating strong cytotoxic effects of SFN. The IC50 value of SFN was found to be 5.7 µM. Various techniques, such as JC-1 staining, the TUNEL test, annexin V–FITC analysis, and AO/PI staining, demonstrated an increase in cell apoptosis following SFN treatment, confirming the occurrence of apoptotic activity. Based on the JC-1 labeling, it is evident that the disruption of mitochondria led to apoptosis, as indicated by the change in the mitochondrial membrane potential. These results were further confirmed through Western blot analysis, revealing changes in the levels of various proteins associated with apoptosis, including p53, Bax, Bcl2, caspase-3, caspase-9, PARP, Smad-4, and cytochrome c. Additionally, the ability of SFN to trigger programmed cell death and halt the cell division process in OECM-1 cells was confirmed through DNA fragmentation and cell cycle analysis. Despite certain limitations, such as the use of a single cell line, and a complete in vitro laboratory setting, the findings demonstrate the potential of SFN as a therapeutic modality for OSCC. The present study forms a foundation for further in vivo and preclinical research, which is necessary to gain a better understanding of the molecular mechanisms and therapeutic effectiveness of SFN in oral cancer treatment.

## Data Availability

The original contributions presented in the study are included in the article, further inquiries can be directed to the corresponding authors.
